# Supplementary material for: Are carbon emissions trading and green financial instruments synergistic? -Comprehensive quantitative research based on content analysis
Source: PLoS One. 2024 Mar 7;19(3):e0298601. doi: 10.1371/journal.pone.0298601 (PMC10919601; doi:10.1371/journal.pone.0298601)
Supplement: S1 Data — (ZIP) [file pone.0298601.s001.zip › Data/地级市Z_F工作报告.zip!/╜¡╬≈/─╧▓2╩╨/└┤╫╘╣1⁄2╓┌║┼í╛┬φ┐╦╩2╛▌═°í┐_2004.docx]

各位代表：

　　现在，我代表南昌市人民政府，向大会作政府工作报告，请予审议，并请各位政协委员和其他列席人员提出意见。

　　一、2003年工作简要回顾

　　过去的一年，南昌是一路风雨、一路艰辛、一路高歌，战胜了突如其来的“非典”、历史罕见的暴雨、百年难遇的高温、前所未有的干旱，实现了招商引资人来客往、经济发展两位增长、社会保障健全有力、科教文卫全面兴旺、三个文明协调发展、社会稳定人民安康，全面完成了市十二届人大四次会议确定的工作任务。

　　国内生产总值641.02亿元，增长15.5%；

　　全社会固定资产投资235亿元，增长67.9%；

　　财政总收入76.5亿元，增长22.9%；

　　地方财政一般预算收入31.4亿元，增长22%；

　　实际利用外资5.83亿美元，增长58.8%；

　　实际利用内资134.88亿元，增长108.0%；

　　社会消费品零售总额201.18亿元，增长13.1%；

　　城市居民年人均可支配收入7793元，增长11%；

　　农民年人均纯收入2808元，增长5.4%；

　　人口出生率13.68‰。

　　（一）经济发展快马加鞭，南昌实力迅速升级。主要经济指标两位数增长，增幅名列全国省会城市前茅。突出表现在：投资需求成倍增长、招商引资增势强劲、工业经济发展迅猛、农村经济蓬勃发展、第三产业繁荣活跃、综合效益更加明显。

　　一是在投资需求上：全社会固定资产投资增幅67.9%，名列全国省会城市第一；贷款余额772亿元，比上年增加156亿元。

　　二是在招商引资上：总额攀升，引资总额名列中部省会城市前茅；结构优化，二产项目实际进资93.5亿元，占全年实际进资额的49.6%；规模扩大，引进超亿元项目65个，其中外资超千万美元的项目26个，内资超亿元的项目39个；全球三大制冷剂制造商之一———格林柯尔投入3.6亿美元，兴建格林柯尔·科龙工业园空调下线，全国最大造纸集团———山东晨鸣纸业一期投入35亿元,打造全国最大的纸业基地正在实施，还有三星奥克斯、东元电机、美国科勒等重大项目也纷纷落户南昌。在橙红橘绿的收获季节，在秋风送爽的美好时日，我们隆重举办了’2003中国南昌第二届金秋经贸活动月。整个活动月做到了有声有色、有板有眼、有模有样、有成有效；迎来了驻华大使、商务参赞，部级领导、五会巨头，专家学者、社会名流，演艺大腕、体育明星。这次活动月，举办大型活动30场、参与人次300万、来昌客商7000多人；伴随着绽放的礼花，满载着丰硕的成果，签订项目206个，其中外资项目36个，引进外资5.35亿美元；内资项目170个,引进内资99.03亿元。

　　三是在工业经济上：朝着打造现代制造业重要基地方向，促进 “园区经济增旺、支柱产业增强、改制步伐增快、上市企业增加”。园区经济增旺：全市12个工业园区入驻工业企业1598个，增长28.2%，实际投产企业1187个；全市工业园区累计完成工业总产值298.3亿元，同比增长38%，实现销售收入73.02亿元，增长34.6%，实现利润18.02亿元，增长39.2%。支柱产业增强：汽车、医药和食品、电子信息和家电、新材料、纺织服装等五大支柱产业，占全市工业的比重超过65%。江铃汽车销售72.94亿元，同比增长28.9%；南飞销售17.1亿元，同比增长2.4%；南烟销售29.83亿元，同比增长12.9%；市政控股产值15亿元，产值增长26.8%，利润增长45.09%；南钢销售42.16亿元，同比增长50%；华源江纺销售5.48亿元，同比增长15.6%；汇仁销售21.73亿元，同比增长41.3%；统一集团销售3.19亿元，同比增长82.2%；桑海集团销售3.2亿元，同比增长35%。南昌已形成商用车、飞机、香烟、饮料、医药、光电子、输液器、视听产品等8个制造业产品基地。改制步伐增快：市直国有（集团）企业147户，已有98户企业基本完成改制。上市企业增加：长力弹簧成功发行5000万A股，募集社会资金3.25亿元，洪城水业已通过中国证监会发审委审核，成为准上市公司。目前，我市有上市公司14家，准上市公司1家，占全省上市公司的58.3%。

　　四是在农村经济上：围绕 “农业增效、农民增收、农村稳定”，推进“三个转移”，即 “土地向大户转移，人口向城镇转移，企业向园区转移”，抓住四个重点：一培养：培养致富带头人；二减少：减少农民负担，减少农村贫困人口；三健全：健全农业支持保护体系、健全农业发展科技支撑体系、健全农副产品市场开拓服务体系；四增加：增加补助、贴息、小额贷款、基础建设投入。促进 “结构优化、规模壮大、机制更新、龙头增加、基础更牢”。结构优化：粮经比例调整为52：48，多种经营占农业总产值比重达82%，经济作物产值比重上升3%，种养比例39.3：60.7，养殖业产值比重上升0.7%。粮食作物优质品率92%，生猪良种率85%，水产品优质品率为30%；规模壮大：建成了特种水产、花卉苗木、无公害水果等8大特色种养示范基地，优质大米、瘦肉型猪、绿色无公害蔬菜等10大优质农产品生产基地。全市已获省级无公害农产品基地36个，共57.5万亩。形成了南昌县水禽、新建县珍珠、进贤县河岸蟹、安义县蔬菜等“一县一业”开发格局；机制更新：稳步推进土地流转，流转土地面积46.5万亩；深化农村税费改革，全年税费总额减幅39.5%，人均减少47.72元，亩均减少31.2元；加大多元化投入力度，全市财政支农资金近2亿元，增长13.6%，争取上级农业资金2.3亿元，实际引进外资2800万美元，实际引进内资8.5亿元，分别是去年的2.9倍和3.5倍；推动农业产业化经营，组建了10个农产品行业协会；龙头增加：市级农业产业化龙头企业50家，省级龙头企业17家，国家级龙头企业3家，年产值超亿元的龙头企业12家，14家龙头企业、30个品牌获国家绿色产品证书；基础更牢：实施国、省道公路改造128.29公里，建成51.76公里，实现沙石路通车76.5公里，全市完成1000公里农村公路改造建设，在全省率先实现乡乡通水泥路；完成退耕还林15.2万亩；全市外洪圩堤建设全面达标，30座病险水库土方达标，治理水土流失面积654公顷，兴建“小山塘”、“小门塘”802座。

　　五是在第三产业上：围绕 “人流畅、货流活、财流旺”，狠抓商贸流通、房地产业、旅游产业三个重点。城中特色街成功打造：中山路、孺子路、胜利路等8条专业街形成特色；城内大商场销售火爆：百货大楼销售13.3亿元，同比增长21.1%，全国排名第25位，洪城大厦销售9.57亿元，同比增长47.3%，全国排名第64位，沃尔玛、丽华大厦、上海联华三个超万平方米的大商场顺利开张（沃尔玛创下单店单日销售额全球第一的业绩）；城郊大市场交易活跃：年交易额超亿元商品市场发展到25个，洪城大市场年交易额108亿元，同比增长6%，在全国同类市场中排名第9位，南昌（深圳）农产品批发市场年交易额26.1亿元，同比增长28.9%，在全国同类市场中排名第7位。房地产业稳步升温：坚持 “控制总量、有序开发、激活市场、促进升值”模式，出台系列政策，激活房屋买卖。全年拍卖挂牌出让土地29宗，计3715.07亩，市本级实现土地经营收入21.22亿元，通过土地储备，新增融资29亿元；新建房屋完成施工面积660.3万平方米，经济适用房42.60万平方米，低洼危旧房改造新建面积7.90万平方米；商品房交易面积330.73万平方米，交易额78.97亿元，同比增长77.86%和120.71%；二手房销售面积156.30万平方米，销售额21.32亿元，同比增长56.27%和75.10%。旅游产业发展强劲：推动 “景点、宾馆、车队、旅行社一条龙，省内省外、境内境外联成网”，强化市场运作，加大资源整合，丰富景区内涵，拓宽投入渠道，开发旅游商品，打响 “五彩缤纷看南昌”品牌，全年游客达到445.74万人次，旅游总收入33.34亿元。

　　六是在综合效益上：全市财政总收入占GDP比重为11.9%，同比提高0.9个百分点；规模以上企业资金利税率为13.97%，同比提高2.2个百分点；经济效益综合指数为139，同比提高14.9个百分点。拥有中国驰名商标2个，省著名商标44个，23个企业30个产品获江西名牌。

　　（二）城市建设展露新颜，南昌品味日益凸现。朝着“五高”目标，高起点规划、高质量建设、高水平管理、高效益经营、高素质塑造；注重“四增”举措，增绿、增亮、增色、增净；从而做大了城市规模，做强了城市功能，做美了城市环境。

　　在城市规模上：一是修订城市规划。规划到2005年，实现城市实际居住人口225万，城市建成区面积230平方公里；规划到2010年，实现城市实际居住人口300万，城市建成区面积300平方公里；规划到2020年，实现城市实际居住人口350万。城市建成区面积350平方公里。二是增加城市人口。出台引才新政，鼓励投资落户，改革户籍制度，降低进城“门槛”。引进高知人才，吸引外来人员，转移农村人口，全市新增城市人口19万人。三是拉开城市格局。一江两岸，南北两城，沿着赣江，两头延伸，张开双翼，敞开四门，内外有环，外带五个组团。昌南：沟通东西南北路，完成防洪包围圈。全年疏散旧城人口20万人，成片拆迁改造低洼地危旧房8万平方米，有效保护民俗街区、风景名胜、革命旧址。昌北：重点是把握三线：梅岭、乌石河、赣江；建好三片：中心区、红角洲、凤凰洲；突出三点：工业园、高校园、人居园。2003年红谷滩新区完成市政道路12条21公里，完成绿化320万平方米，完成拆迁48.06万平方米，完成征地1.2万亩，启动了云溪、凤凰、红岭、龙岗卫东、丰和6大花园小区110万平方米还建房建设。

　　在城市功能上：一是畅通城市交通：重点是打通出城大路、改造城中道路、修建环城高速路、整修街道小巷路。出城大路：推进了6条对外道路建设，完成投资近4亿元。其中瑶湖大道道路已完工，9公里昌进公路一期春节前完成整幅道路，29.5公里省大公路、29.1公里南安公路完成半幅道路，实现通车，红湾公路已开工，南高公路正在抓紧拆迁；城中道路：开工建设26条，完成投资近10亿元，其中昌东大道、洪都南大道、洪都北大道、高新北大道、北京西路已完工，解放东路、北京东路正在加紧建设，庐山南大道绿化、亮化已经完成；环城高速：东外环高速已经开始拆迁工作，西外环高速正在进行前期准备；街道小巷路：实施“百路大会战”，投入资金1亿多，改造道路109条。二是构建物流平台：保税仓库，已纳入昌北物流规划；无水港，已和宁波海关协商；南昌国际集装箱码头，全面启动；昌北国际机场，已被批准；海关办事处，抓紧进行；铁路西站、中央商务区，正在规划之中。三是强化基础设施：重点是学校医院、公交线路、公共建筑、体育场馆、供水供电、煤气燃气、污水处理。配合新建高校园区2个，改造中小学危房12万平方米；新增公交线路8条；竣工公建项目510项，建筑面积545.7万平方米；全面铺开青山湖体育公园建设；完成青云水厂三期工程；新建2座变电站、安装市内变压器130台；启动红谷滩新区液化气供应项目；完成青山湖污水处理厂土方工程，全面展开土建安装。四是搞好市政配套：重点是增设地下管网，城市排水雨污分流工程全面开工；规范空中线网，电线、电话线、电视线、电缆线逐片规范；扩充公共绿地，全年扩充公共绿地244公顷；兴建市民广场，建成上营坊社区休闲广场、福州路休闲广场，免费开放人民公园。

　　在城市环境上：重点是装点水面，打造景点，注重“四增”。一是装点水面。南昌是江南水乡，滨江依湖。古代是：七门九洲十八坡，三湖九津通赣鄱；现在是：赣江穿城而过，东西有两河，内外有八湖；可谓是：城在湖中，湖在城中。要做好山水文章，发挥水城优势，就必须重点打造：一江两河八湖。赣江两岸：一桥在启动、三桥要贯通。启动了生米大桥：该大桥投资4.8亿元，2005年建成后，犹如：鲲鹏两翼、气势磅礴；兴建了前湖电排站：该电排站6台机组，3000千瓦，投资3000万元；竣工了秋水广场：该广场长1100米，宽110米，面积8万平方米，水柱128米。赣江要打造：站在东岸看西岸，站在西岸看东岸，乘坐游船看两岸。现在已呈现：在东岸，登临滕王高阁：远眺西山积翠，中观红谷新府，近看秋水喷泉；在西岸，漫步秋水广场：静观章江晓渡，领略滕阁秋风，纵览南浦飞云；泛舟江中看两岸：淼水季节，白天汹涌澎湃，晚上涛声依旧；常水时节，白天碧波荡漾，晚上万点星光；百年难遇的枯水季节，仍然是白天水天一色，晚上灯火辉煌。抚河两岸：现在是家家装修，店店美容，人来客往，车水马龙，繁华似锦，商机兴隆。玉带河：嘴上说了39年，图上画了5年，现在干了1年4个月。这条河穿过京九线，跨过9条大道，大小桥梁20座，拆迁面积30万平方米；地上拆迁千磨万难，地下管线千丝万缕，拆迁难度、施工难度前所未有；2004年我们要求春节桥通车，五月水分流，年底河露脸；真正使这条河体现一要亲水、二要休闲、三要自然、四要文化内涵，做到上看上好看、下看下好看、坐船游河更好看。内四湖：装点四湖，整合“四名”，打造城市瑰宝、南昌明珠。展示杏花名楼之毓秀、娄妃梳妆之余韵；重现晨钟暮鼓、东湖夜月、苏圃春蔬、徐亭烟柳之胜景。青山湖：前期重规划、截污流、清淤泥、换活水，后期兴建环湖一路，打造东西南北各一园（燕鸣岛公园、相思林公园、湖滨公园、丹霞公园），要展现：白天胜似西湖，晚上恰如彩虹。象湖：整体规划分步实施。已完成环湖一路、湖心四岛、灌婴广场、观礼台、万寿宫、藕香榭、环漪阁、渡口长亭；正在施工的有沙滩泳场、垂钓中心；届时将展示：一面荷花四面柳、一城春色半城湖，亭台楼阁、俊彩星驰，金色沙滩、温馨港湾，江南水乡尽在象湖，白天流连忘返，晚上灯火阑珊。二是打造景点。南昌有着2200多年的历史文化。只有挖掘文化底蕴，打造新的景点；才能传承豫章悠久文化，再现洪都风土人情。绳金塔：完成了“一塔一寺一庙一村一园一院”，要使之成为“南京的夫子庙、上海的城隍庙”，让游客听南昌话、看南昌戏，尝南昌菜、品南昌趣。万寿宫：迁址象湖之滨、两易寒暑告成。占地70余亩，已经完成五殿二楼二门头；这正是一湖春色一重天、碧波浩淼柳如烟，秉承豫章风情谱、重建古观思前贤。梅湖：展示豫章城南之十景，弘扬画派宗师之才情，传承净明道派之忠孝，再现田园风光之胜景。革命纪念地：八一起义总指挥部、朱德旧居、贺龙指挥部、叶挺指挥部、军官教育团、新四军军部旧址等革命历史遗存，严格保护、严格控制，运用现代科技强化硬件，充实时代内涵丰富软件，从而营造庄严气氛、弘扬革命精神。三是注重“四增”。增绿：新铺草面积138万平方米，新栽乔木42.8万株、灌木956.7万株，绿地覆盖率36.6%，绿化率34.8%，人均绿地7.1平方米；增亮：打造灯光点、构筑灯光群、营造灯光带、新增路灯6.18万盏；增色：穿衣戴帽、一路一色、一路一景、色彩协调、轮廓清晰；增净：重点改善天上的空气质量，改变地面的市容市貌，改优江河湖泊的水质。空气质量由Ⅲ级上升为Ⅱ级，区域噪声下降0.10分贝；改造公厕64座,新增公厕14座，垃圾无害化处理率达到100%；整治污染企业147家，关停11家，实行了医疗废弃物集中处置，建设了青云水厂饮用水源水质自动监测站。

　　（三）现代文明好戏连台，南昌经验名动全国。推进五大文明工程，优化社会发展环境，加快民主政治建设，全力维护社会稳定，协调发展各项事业。推进五大文明工程：按照 “有板有眼、有声有色、强力推进、不断深入”要求，全面实施 “文明行风、文明表率、文明市民、文明社区、文明村镇”五大工程。文明行风工程，突出群众、企业、县区、上级“四满意”；文明表率工程，突出决策方式、思想道德、从政行为、社会行为“四文明”；文明市民工程，突出讲礼貌、讲法纪、讲科学、讲卫生；文明社区工程，突出依法治市、环境卫生、文体活动、社会稳定；文明村镇工程，突出改善生活环境、改善生活条件、改善生活质量。重点开展行风评议评价、创先争优、道德标兵评比、文明市民标兵评比、“星级社区创建”、百村文明示范、千村环境整治等活动。南昌“以创建促发展、以发展促创建”的经验，被中央宣传部、文明办，赞誉为“南昌模式”，并组织11家中央级主流媒体聚焦南昌，30多名资深记者聚集南昌，进行了为期一周的专题采访，新闻联播头版头条连播3次，发出新闻报道40多篇，全方位多视角向全国隆重推出南昌。优化社会发展环境：重点是再减审批事项、畅通办证通道、领导亲自服务、追究落到人头。审批：再减16.4%，仅保留347项，为全国最少的城市之一；办证：建立外商投资绿色通道，一站式服务，限时办结；服务：开通经济热线110，解决问题全天候；追究：严格落实外商投诉事项，外商投诉解决率达100%。同时，加强政、银、企合作。可以说，优化投资环境服务年活动推动的33个项目项项已落实、项项有成效。去年世界银行评定：南昌投资环境为B+类，列全国第11位，与北京、深圳同档。加快民主政治建设：机构改革：乡镇个数由101个精减为80个，精减20、8%；乡镇行政编制由3465名精减为2669名，精减23%；换届选举：村委会1172个、居委会258个全面换届；廉政建设：查办大案要案28件，出台廉洁自律制度7项，颁发治理腐败源头制度10项；法制建设：提请市人大常委会审议地方性法规草案10件，制定规章2件，清理规章69件，办理规范性文件备案26件，办理行政复议案件58件。认真执行人大及其常委会的决议，自觉接受人大法律监督、工作监督和政协民主监督。全力维护社会稳定：坚持“稳定无小事，没事当有事，小事当大事，有事不怕事”方针，在社会治安上：建立社会治安防控体系，强化110，加强派出所，控制社会面，提高破案率。破案率超过40%，街面刑事案件下降13%；在安全生产上：坚持了警钟长鸣、排除隐患、责任到人，杜绝了重大恶性事故和特大事故，做到了“多打铁少打人”；在消防安全上：要求大喊大叫、大排大查、大整大改、大添大建、大操大练，全年未发生重特大火灾事故；在信访工作上：领导高度重视，部门解决问题，信访总量稳中有降，越级集访明显减少。在关爱“弱势群体”上：站在“立党为公、执政为民”的高度，坚持“以人为本、关爱百姓、带着感情、饱含深情”，做到：“一枝一叶总关情”。重点帮助下岗职工、城镇低保户、农村特困群众三类群体。对下岗职工：实施一个工程，做到两个确保。一个工程：64331216工程。即：新增就业人员6万人，安置下岗失业人员4万人，安置“4050”人员3千人，安置双下岗人员3千人，安置城镇困难人员1千人，安置残疾人就业2千人，转移农村劳动力16万人。举措是：搭好28个社区平台；落实九大方面优惠政策（税费减免、小额贷款、社保补贴、就业援助、主辅分离、就业服务、财政投入、企业裁员、社区平台）；发放2000万元小额贷款。政府追求的目标是：尽量使职工分流不回家，鼓励职工积极争取，下岗就业创辉煌。全市共安置城镇就业人员6.5万人，其中下岗失业人员再就业4.2万人。两个确保：下岗职工基本生活费、离退休人员养老金100%按时足额发放。全市共发放：下岗职工基本生活费9000万元，离退休人员养老金11亿元。对城镇低保户：扩大覆盖面（人数由8.5万人增加到10万人，同比增加18%），提高标准额（市由143元提高到165元；县由104元提高到125元），做到应保尽保。全市共发放低保资金6161万元，比去年增加3135万元。对农村特困群众：加大扶助力度，保障基本生活，做到摸清户头，落到人头，雪中送炭，疏而不漏。全市共发放社会救助资金2403万元，比去年增加970万元。协调发展各项事业：实施三名工程，推动教育发展。创名校10所、建名科10个、授名师10人；小学、初中入学率为99.4%和97.1%，高考录取率为79.65%。推进科技改革，构建创新体系。认定市级制造业信息化示范企业12家、市级科技示范企业10家、培育优质健康农产品科技示范基地6家。实施人才战略，构筑人才高地。确立“人才绿卡”制度，出台引进海内外优秀人才办法，建立首家博士后工作站，实施“百千万”人才工程。加强疾病控制，健全卫生体系。疾病控制得到加强、卫生网络逐步健全、服务水平继续提高。特别是在“抗非”斗争中，全市上下着眼防、全力控、立足治，实行村自为战、户为基础、洁身自保、把住关口、部门到位、责任到人，做到天罗密布、地网层设。南昌抗击“非典”实现“双零”记录（无一例“非典”病人、无一例疑似病例），在中部省会城市一枝独秀，确保了南昌“一方净土、一片蓝天”。成功举办“四进社区”文艺展演，首次获得“五个一工程”大奖。第二届全国“四进社区”文艺展演，31个省市400多名参演人员云集南昌，展演节目短小精悍、形式多样，展示社区建设新成果、社区文明新气象、社区居民新风尚。广播剧《马克思的一天》以精深的思想、精湛的艺术、精良的制作，使南昌首次拿下“五个一工程奖”，实现“零的突破”，终圆“十年之梦”。城运会上再创佳绩，群众体育日益普及。在第五届全国城运会上，南昌健儿不畏强手，顽强拼搏，捷报频传，凯旋而归，喜得金牌6枚、银牌8枚、铜牌1枚，跻身全国省会城市前10强。加大人口与计生工作力度，圆满完成省下达目标任务。同时，加强全民国防教育，促进民兵、预备役、人民防空建设；加强军民共建，荣获全国双拥模范城“五连冠”；加强对外联络，与广州市缔结为友好城市；加强外事、侨务、对台、民族、宗教、参事、档案、史志、老龄和工会、共青团、妇女、儿童、残疾人等工作。

　　各位代表：过去的一年，南昌是好事连篇、好戏连台、好歌连唱。上述成绩的取得，归功于党中央、国务院方针政策的指引，归功于省委、省政府的正确领导，归功于省直部门的大力支持，归功于社会各界的热情帮助，归功于全市上下的共同努力。在此，我谨代表南昌市人民政府，向为南昌经济发展和社会进步做出贡献的全市工人、农民、干部和知识分子，致以崇高的敬意！向各民主党派、工商联、群众团体、社会各界朋友和驻昌解放军指战员、武警官兵和广大公安干警，表示衷心的感谢！

　　当然，我们在前进中还存在不少困难和问题，主要是：经济总量仍然不大；综合实力仍然不强；服务水平仍然不高；就业压力仍然较大；不稳定因素仍然不少。对此，我们必须高度重视，采取有效措施，切实加以解决。

　　二、当前面临的形势和2004年政府工作任务

　　2004年是贯彻落实党的十六届三中全会精神、全面建设小康社会的关键之年。面对东部大发展、西部大开发、中部大崛起、东北大振兴的发展环境，我们必须乘势而上，一鼓作气，合力争先。

　　2004年政府工作的总体要求是：

　　认真贯彻党的十六届三中全会精神，全面践行“三个代表”重要思想，始终坚持“五个统筹”方针，牢固树立全面、协调、可持续发展观，按照市委八届六次全会确定的工作思路，坚持开放为先、改革跟进、城建加速、稳定保障、协调发展的工作方略，坚持以工业化为核心，以大开放为主战略，以机制体制创新为动力，走工业化、城市化、农业现代化“三化”互动的路子，团结和带领全市人民，全力打造一个基地：现代制造业重要基地；做好两篇文章：建设现代区域经济中心城市，建设现代文明花园英雄城市；确保三个率先：在全省率先实现工业现代化，率先建成现代文明花园城市，率先全面实现小康社会；实现四个提升：提升城市活力，提升经济实力，提升城市品位，提升现代文明；为做大做强南昌、实现“富民强市”目标而努力奋斗。

2004年主要预期目标是：

　　国内生产总值730亿元，增长14%；

　　财政总收入88亿元，增长15%；

　　地方财政一般预算收入35.5亿元，增长13%；

　　全社会固定资产投资300亿元，增长28%；

　　实际利用外资6.6亿美元，增长13%；

　　实际利用内资160亿元，增长18.6%；

　　社会消费品零售总额227亿元，增长13%；

　　城市居民年人均可支配收入8400元，增长7.8%；

　　农民年人均纯收入3000元，增长6.84%；

　　人口出生率控制在14‰以内。

　　（一）开放为先，提升城市活力

　　坚持大开放，开展大招商；对接长珠闽，融入全球化。开展大招商。继续围绕“开放目标更高、开放领域更宽、开放力度更大、开放优势更强”要求，诚招天下客，善待来昌人。着重做到“四个突出”：突出瞄准招商重点。瞄准重点地区、重点企业、重点人物，力争引进世界500强企业1-2个，国内200强企业2-3个；突出发挥主战场作用。力争高新开发区、经济开发区、民营科技园三个园区，在全国同类园区位置前移；突出改进招商手段。开展项目招商、网上招商、会展招商、驻点招商、代表招商、中介招商；突出改善投资环境。打造服务型政府，建立信用型南昌。优化招商环境，提高办事效率，严格落实外商代办绿色通道，切实帮助解决困难问题，抓好重大项目跟踪落实。对已签约项目，严格“四定两保”，即定时间、定目标、定责任、定奖惩，保证按时开工、保证按时投产。与此同时，加大财政、税收扶持力度，用足用好出口退税政策，加大制成品的出口比重。对接长珠闽。切实做到“三个对接”：法规政策对接。凡“两洲一区”已取消的行政审批事项，原则上对应取消；体制机制对接。按市场规律办事，按法律法规办事，管住管好该管的事，放开放活不该管的事；要素市场对接。促进资本、土地、劳动力、技术、信息等要素市场，对接“长珠闽”，融入全球化。与此同时，朝着使国有、民营、混合所有制经济竞相发展目标，基本完成国企改制，大力发展非公有制经济，加快企业上市步伐。

　　国有企业：加快大中型企业建立现代企业制度步伐，加快中小企业放开搞活步伐。改制企业原欠市、县（区）本级财政预算安排的支工、支农、支商资金，经债权同级财政部门审定后报政府批准予以核销。力争2004年基本完成国有企业改制。非公有制企业：拓宽融资渠道，用好中小企业发展基金，力争非公有制经济“五分天下有其二”。企业上市：力争洪城水业第一季度发行上市，募集社会资本2.5亿元；行知软件上半年在香港创业板成功上市；力争先锋软件通过中国证监会发行审核；龙鹏股份、江联环保等企业进入上市辅导期。

　　（二）五业并举，提升经济实力

　　坚持让工业、农业、商贸三产、个私民营齐头并进，让劳动、知识、技术、管理、资本竞相迸发；围绕全力打造现代制造业重要基地，壮大经济总量，提升经济质量。工业突出：改制、改组、改造。改制：调动内因、凝聚内气、盘活内资；改组：引进外资、借助外力、激活外钱；改造：技改贴息、挖掘潜力、打出品牌。农业突出：调整、优化、提高。调整：调优结构、调大规模、调出特色；优化：优化布局、优化品质、优化生产方式；提高：提高科技含量、提高附加值、提高商品率。商贸三产突出：提升、发展、拓宽。提升传统商贸，发展新型业态，拓宽现代服务业。个私民营突出：放开、平等、服务。放开：降低投资门槛；平等：待遇一视同仁；服务：搭好服务平台。具体措施是：加大投入强度，做强园区经济，培植优强企业，形成支柱产业。在投入强度上：要坚持上级资金、社会资金、信贷资金、企业资金、民间资金五管齐下，重点是争取国家资金、用好信贷资金、激活民间资金，确保投资对经济的贡献率达50%以上。在园区经济上：工业园区要做到：功能完善、错位发展、集约经营。功能完善重点是：运用市场手段，加快基础建设，确保“四通一畅”；错位发展重点是：扬长避短、扬优成势。南昌高新开发区重点发展电子与信息、光机电一体化、新材料、生物医药产业，南昌经济开发区重点发展汽车、机械制造等产业，民营科技园重点发展食品和保健食品、服装等产业；集约经营重点是：控制用地规模、核准投入产出、衡量纳税金额，推动工业园区轴线延伸，网状扩展。农业示范园要做到：市场运作、注重特色、带动农户。市场运作重点是：面向市场找订单、扶龙头、帮协会、融资金、深加工、产业化；注重特色重点是：立足现有优势、创出自身特色。扬子洲现代生态农业科技园重点发展无公害蔬菜，南昌高梧现代农业科技示范园重点发展生猪、花卉，桑海、小兰、进贤农业科技园重点发展，农副产品加工；带动农户重点是：依托园区拓展市场、组织农户壮大规模。市级重点扶持发展扬子洲、高梧、黄洲三大农业科技示范园。现代物流园要做到：合理规划、培育龙头、功能配套。合理规划重点是：规划昌南、昌北现代物流园，论证墨山粮食（食品）大市场、市果品交易市场、再生废旧市场；

　　培育龙头重点是：吸引一批物流企业进入园区、扶持一批企业搞活物流；功能配套重点是：推行采购配送、电子商务，发展连锁经营、新型业态；总之，各类园区经济都要在政策上，对接“长珠闽”、融入全球化，使园区真正成为经济技术聚集区、科技进步先导区、体制改革实验区，成为南昌经济的增长极、支撑点。在优强企业上：一是争取政策。争取国家项目立项，争取国家债券资金，争取省里技改贴息，力争全年投入20亿元；二是扶持龙头。工业重点扶持：江铃、南烟、南钢、江中、泰豪科技、诚志股份、济民可信、汇仁药业、正邦集团、华春集团、恒大集团等；农业重点扶持：阳光乳业、国鸿公司、汪氏蜂蜜、煌上煌集团、桑海集团、圣丰兔业、南昌（深圳）农产品批发市场等；商业重点扶持：百货大楼、洪城大厦、洪城大市场、亨得利、新洪客隆等。三是协作配套。坚持“政府引导、市场运作、杠杆撬动”，以工业园区为主战场、以重大项目为中心、以企业为主体，加强专业化协作，实施专业化配套，力争全年实施配套项目50个，新增配套资金50亿元以上。四是优化结构。全力抓好江铃汽车、晨鸣纸业、华源江纺、润田果汁等技改项目。稳步推进“土地向大户转移、人口向城镇转移、企业向园区转移”步伐，实现2004年粮食作物优质品率达95%，生猪良种率达88%，水产品优质品率达35%，农产品商品销售率达76%，城镇化率达47%。大力实施退耕还林工程，深化粮食流通体制改革，建立市级粮油储备，加强农产品质量安全检验检测，确保农产品供应安全。在支柱产业上：工业重点壮大汽车、医药和食品、电子信息和家电、新材料、纺织服装五大产业；农业重点培育翘嘴鳜、淡水珍珠、花卉苗木、无公害蔬菜、食用菌菇、小水果、河蟹、荞头、奶牛、藜蒿十大基地；三产突出商贸流通、房地产、旅游三个重点，实现社会消费品零售总额22亿元、房产交易总额100亿元、旅游业总收入50.43亿元。

　　（三）规、建、管并重，提升城市品位

　　一个城市规划建设管理如何，标志着这座城市的整体形象，反映着这座城市的领导者水平，体现着市民的文明程度。南昌要描绘历史画卷、勾画世纪蓝图、奠定千秋伟业，就要坚持人性化、个性化、灵性化、中心化四个原则，创造发展兴奋点、引导投资新热点、培植经济增长点、打造都市风景点。具体举措是：拉开城市框架、着力城市经营、完善城市功能、加强城市管理、美化城市形象。在城市框架上，主要是：根据交通战略规划，按照“东西展两翼、四门齐畅通、南北各一环、中间套一环”的要求，构筑城市路网系统：启动城市外环、改造城市中环、打开出城通道、建设环城高速。城市外环：完成昌东大堤、拓宽胡惠元堤、城南隔堤。城市中环：主要完成解放路改造。出城道路：围绕全力打造半小时经济圈，向东：完成北京东路改造；向南：加快改造迎宾大道，建设南昌至进贤一级公路；向西：加快建设红湾一级公路，新建昌湾大道、拓宽省大一级公路；向北：完成建设新祺周至安义一级公路。环城高速：抓紧乐温高速公路建设，启动西环城高速公路；同时，加快国际集装箱码头项目建设，抓紧生米大桥建设。在城市经营上，主要是：严格遵守法律法规，严格执行审批程序，强化土地储备中心功能，精心运作三大公司（土地经营公司、城建投资公司、市政投资公司），有效盘活三块资源（城市国有土地、城市基础设施、城市无形资产），力争全年土地出让金突破35亿元。在城市功能上，主要是：加强城市南、北防洪，畅通供水排水系统，完善供气供电设施，健全交通信息系统，推进小城镇配套建设，改造乡村供水和道路。城市防洪：实施富大有堤（高新大道至鱼尾闸）6.9公里大堤除险加固工程，构筑昌南城区百年一遇外防洪包围圈；实施红角洲生米段抽沙造地工程，建设昌北赣江大道（生米大桥至鱼目山）14.59公里百年一遇标准防洪堤；完成20座重点小型以上水库除险加固及达标建设，完成泵站的维修更新及达标建设。供水排水：完善青云水厂三期工程后期建设，新建牛行水厂；加快改造玉带河、桃花河和青山湖东、西渠，全面整治旧城区积水，实施城市地下管线同沟共井工程。供气供电：完善城南、城东现有人工管道煤气供气，加快朝阳地区、红谷滩新区管道液化气建设；加快新建城区8座变电站及输配线路建设，推动老城区居民低压电网改造。交通信息：新建洪城大市场停车场等5个大型公交场站，新、改建南昌长途客运站等7个客运站，启动7个货运站建设；继续建设城乡宽带信息网络，搭建公共信息共享平台。小城镇：加快建设向塘、民和、龙津3个次中心城镇，温圳、李渡、文港、西山、石鼻、招贤等14个中心镇（重点镇），努力形成各具特色的大中小城市一体化发展的格局。乡村供水和道路：继续兴建“小山塘”、“小门塘”，彻底解决偏远乡村“人无水用、牛无水饮、田无水灌、火无水救”的问题；继续加大农村公路硬化改造力度，彻底改变偏远乡村“先大车后小车、最后扁担加土车”的交通状况。在城市管理上，主要是：继续整治市容环境，不断强化交通管理，抓好废水、废气、废物治理，加强环境监测控制，提高建设施工水平。市容环境：进行第二轮“百路大会战”，新建和改造一批环卫设施。交通管理：强化交通管理，规划建设城市停车场。废水、废气、废物治理：切实解决小街小巷、插花地带的脏乱差现象；对大气、水污染物实施总量控制，推行排污许可证制度。环境监测控制：严格实行“三同时”制度和环境影响评价制度；完成大气质量自动监测四个子站、饮用水源自动监测四个子站、环境综合监控系统、医疗废物处置中心的建设。建设施工：注重工程质量、做到安全生产、创建文明工地。在城市形象上，主要是：体现江南水乡气质、坚持“四脉”合为一体的要求，突出把握昌北新建新城展新貌、昌南拆建旧城换新颜，彰显“一江两岸、双城拥江”特色；继续抓好绿化、美化、亮化、净化工程，抓好水资源和土地资源保护工程，抓好一江两河八湖整治，新建12个公园、16个休闲广场，绿化覆盖率达到37%。今年：政府投融资15亿元，工程项目5大类，好事实事87件。总之，我们要把好“四条脉”（人脉、文脉、山脉、水脉），写好“八个字”（一张图、一条路、一支水、一盏灯、一棵树、一栋楼、一条线、一篇文），展现南昌“蓝天常现、碧水常流、绿色长驻、春色满城”城市魅力。

　　（四）以人为本，提升现代文明

围绕争创“四城”目标：国家卫生城，全国文明城，国家环保模范城，全国园林城。深入推进五大文明工程，协调发展各项社会事业，全力维护社会秩序稳定，不断提高人民生活水平，促进经济社会可持续发展。五大文明工程，重点是：切实做到：规范跑马路的（闯红灯、越黄线、鸣喇叭、开大灯），教育逛马路的（街上乱窜、口里乱吐、手里乱扔、护栏乱爬），提高站马路的（过去：打桩、砌墙、拉网；现在：装灯、划线、处罚），褒奖扫马路的，讲好话、办好事、做好人。从而：打出英雄城品牌，塑造南昌人形象。各项社会事业，重点是：创新科技体制、构筑人才高地、优先发展教育、繁荣文化广电、发展卫生事业、提高体育水平。科技体制：建立企业为主、院所参与、部门支持技术创新体系、多元化科技投融资体系和科技中介服务体系。人才高地：坚持党管人才原则，引进高层次人才，培养高素质人才，发展实用型人才。教育：优化义务教育网点布局，做大做强高中阶段教育，整合提升职业教育资源，努力打造高等教育品牌。启动南昌二中、南昌十九中新校区建设，完成南昌科技大学一期工程。文化、广电：挖掘文化底蕴，发展文化产业，丰富文化生活，打响文化品牌。启动铁军广场，加快红谷滩文化宫、广电中心、报业大厦建设。卫生：健全公共卫生体系，完善卫生网络建设，加大卫生执法力度，加强农村卫生保健水平。启动市疾控中心、江西南昌紧急救援中心建设。体育：广泛开展群众性体育运动，不断提高竞技体育水平，抓紧启动国际体育中心建设。社会秩序稳定，重点是：加强民主法制。加强行政立法，严格依法行政，强化行政监察和层级监督，促进廉政勤政建设，推进法制化投资环境建设。自觉接受人大法律监督和工作监督、政协民主监督和社会舆论监督，认真办理人大代表议案、建议和政协提案，重视发挥各民主党派、工商联、各群众团体作用。确保三项安全。加强社会治安综合治理，严厉打击犯罪行为。坚持“安全第一、预防为主”，加强安全生产，确保消防安全。增强国防观念、强化忧患意识、完善人防体系、加强信访工作。提高人民生活水平重点是：强化就业保障，推进民心工程。在就业保障上：一是提高就业比率。安置城镇劳动力5万人，安置下岗职工1.5万人，转移农村劳动力16万人，培训技工4万人；二是强化保障体系。建立健全养老保险、失业保险、医疗保险三项体系，2004年各项社会保障基金收缴率达到90%。大力发展慈善事业，拓宽资金筹集渠道；三是扩大低保覆盖面。尤其是对农村五保户，要摸好底、造好册、上好网，做到：规范化管理，实现应保尽保；四是健全救助机制。属地管理、分级负责，确保特困人口职工基本生活费。加大农民工资清欠力度，切实维护农民务工权益；五是实现两个确保。下岗职工基本生活费和企业离退休人员养老金100%发放。在民心工程上：突出抓好就业再就业工程、食品药品放心工程、农村扶贫开发工程、农村道路工程等20件实事，切实做到“情为民所系、利为民所谋”。可持续发展，重点是：综合治理人口问题、稳定低生育水平；落实资源保护政策，合理开发自然资源；不断改善环境质量，促进人与自然和谐发展。

　　总之，南昌要以开放提升城市活力、以经济提升城市实力、以环境提升城市品位、以文明提升城市形象。使南昌的经济规模和城市规模迅速做大，使南昌的经济实力和承载能力全面做强。

　　各位代表，讲历史———南昌风光无限，讲现在———南昌商机无限，讲未来———南昌前景无限。让我们紧密团结在以胡锦涛同志为总书记的党中央周围，乘十六届三中全会东风，全面践行“三个代表”重要思想，在市委的正确领导下，为了440万人民，发动440万人民，带领440万人民，敢作敢为，善作善为，为做大做强南昌、实现富民强市，策马扬鞭，高歌猛进！
